# Community Engagement in Vaccination Promotion: Systematic Review and Meta-Analysis

**DOI:** 10.2196/49695

**Published:** 2024-05-10

**Authors:** Yao Jie Xie, Xiaoli Liao, Meijuan Lin, Lin Yang, Kin Cheung, Qingpeng Zhang, Yan Li, Chun Hao, Harry HX Wang, Yang Gao, Dexing Zhang, Alex Molassiotis, Gilman Kit Hang Siu, Angela Yee Man Leung

**Affiliations:** 1 School of Nursing The Hong Kong Polytechnic University Hong Kong China (Hong Kong); 2 Research Centre for Chinese Medicine Innovation The Hong Kong Polytechnic University Hong Kong China (Hong Kong); 3 Musketeers Foundation Institute of Data Science The University of Hong Kong Hong Kong China (Hong Kong); 4 Department of Pharmacology and Pharmacy LKS Faculty of Medicine The University of Hong Kong Hong Kong China (Hong Kong); 5 School of Public Health Sun Yat-sen University Guangzhou China; 6 Usher Institute Deanery of Molecular, Genetic & Population Health Sciences The University of Edinburgh Edinburgh United Kingdom; 7 Department of Sport, Physical Education and Health Hong Kong Baptist University Hong Kong China (Hong Kong); 8 JC School of Public Health and Primary Care The Chinese University of Hong Kong Hong Kong China (Hong Kong); 9 Health and Social Care Research Centre University of Derby Derby United Kingdom; 10 Department of Health Technology and Informatics Faculty of Health and Social Sciences The Hong Kong Polytechnic University Hong Kong China (Hong Kong); 11 Research Institute on Smart Aging (RISA) The Hong Kong Polytechnic University Hong Kong China (Hong Kong)

**Keywords:** community engagement, community-based participatory research, vaccination rate, health promotion, vaccine.

## Abstract

**Background:**

Community engagement plays a vital role in global immunization strategies, offering the potential to overcome vaccination hesitancy and enhance vaccination confidence. Although there is significant backing for community engagement in health promotion, the evidence supporting its effectiveness in vaccination promotion is fragmented and of uncertain quality.

**Objective:**

This review aims to systematically examine the effectiveness of different contents and extent of community engagement for promoting vaccination rates.

**Methods:**

This study was performed in accordance with the PRISMA (Preferred Reporting Items for Systematic Reviews and Meta-Analyses) guidelines. A comprehensive and exhaustive literature search was performed in 4 English databases (PubMed, Embase, Web of Science, and Cochrane Library) and 2 Chinese databases (CNKI and Wan Fang) to identify all possible articles. Original research articles applying an experimental study design that investigated the effectiveness of community engagement in vaccination promotion were eligible for inclusion. Two reviewers independently performed the literature search, study selection, quality assessment, and data extraction. Discrepancies were resolved through discussion, with the arbitration of a third reviewer where necessary.

**Results:**

A total of 20 articles out of 11,404 records from 2006 to 2021 were retrieved. The studies used various designs: 12 applied single-group pre-post study designs, 5 were cluster randomized controlled trials (RCTs), and 3 were non-RCTs. These studies targeted multiple vaccines, with 8 focusing on children’s immunization, 8 on human papillomavirus vaccine, 3 on hepatitis B virus vaccine, and 1 on COVID-19 vaccine. The meta-analysis revealed significant increases in vaccination rates both in pre-post comparison (rate difference [RD] 0.34, 95% CI 0.21-0.47, *I*^2^=99.9%, *P*<.001) and between-group comparison (RD 0.18, 95% CI 0.07-0.29, *I*^2^=98.4%, *P*<.001). The meta-analysis revealed that participant recruitment had the largest effect size (RD 0.51, 95% CI 0.36-0.67, *I*^2^=99.9%, *P*<.001), followed by intervention development (RD 0.36, 95% CI 0.23-0.50, *I*^2^=100.0%, *P*<.001), intervention implementation (RD 0.35, 95% CI 0.22-0.47, *I*^2^=99.8%, *P*<.001), and data collection (RD 0.34, 95% CI 0.19-0.50, *I*^2^=99.8%, *P*<.001). The meta-analysis indicated that high community engagement extent yielded the largest effect size (RD 0.49, 95% CI 0.17-0.82, *I*^2^=100.0%, *P*<.001), followed by moderate community engagement extent (RD 0.45, 95% CI 0.33-0.58, *I*^2^=99.6%, *P*<.001) and low community engagement extent (RD 0.15, 95% CI 0.05-0.25, *I*^2^=99.2%, *P*<.001). The meta-analysis revealed that “health service support” demonstrated the largest effect sizes (RD 0.45, 95% CI 0.25-0.65, *I*^2^=99.9%, *P*<.001), followed by “health education and discussion” (RD 0.39, 95% CI 0.20-0.58, *I*^2^=99.7%, *P*<.001), “follow-up and reminder” (RD 0.33, 95% CI 0.23-0.42, *I*^2^=99.3%, *P*<.001), and “social marketing campaigns and community mobilization” (RD 0.24, 95% CI 0.06-0.41, *I*^2^=99.9%, *P*<.001).

**Conclusions:**

The results of this meta-analysis supported the effectiveness of community engagement in vaccination promotion with variations in terms of engagement contents and extent. Community engagement required a “fit-for-purpose” approach rather than a “one-size-fits-all” approach to maximize the effectiveness of vaccine promotion.

**Trial Registration:**

PROSPERO CRD42022339081; https://www.crd.york.ac.uk/prospero/display_record.php?RecordID=339081

## Introduction

Vaccination stands as one of the top 10 great public health achievements of the last century. It has made significant strides in eliminating and controlling various vaccine-preventable diseases, as evidenced by the reduction in morbidity, mortality, and disability caused by these diseases [1,2]. A notable illustration is the use of vaccines as a crucial measure to mitigate the COVID-19 pandemic in the past 3 years [3,4]. A previous study analyzed the economic advantages of vaccination against 10 diseases across 73 countries from 2001 to 2020. It reported that vaccinations have prevented over 20 million deaths and saved approximately US $350 billion in disease costs [5]. A modeling study examined the health implications of vaccination against 10 pathogens across 98 countries from 2000 to 2030. It revealed that vaccinations have prevented 69 million deaths [6].

Both the Global Vaccine Action Plan 2011-2020 and Immunization Agenda 2030 have established strategic objectives to immunize every eligible individual with appropriate vaccines and to ensure equitable coverage of immunization benefits for all. However, the immunization coverage of many vaccines has yet to reach the expected level. For instance, between 2006 and 2014, only 47 million women across 80 countries and territories received the full course of human papillomavirus (HPV) vaccines, representing a mere 1.4% coverage of the total female population [7]. In addition, a study assessing the coverage of childhood vaccines across 1366 administrative regions in 43 countries revealed that only one-third of children in 4 countries had fully received routine childhood vaccines [8]. In terms of adult vaccination, only 11 out of 204 countries achieved the World Health Organization (WHO) target of 90% coverage for 11 routine vaccines by 2019 [9]. Various reasons and barriers contribute to the lack of vaccination, with a significant obstacle being vaccine hesitancy. Vaccine hesitancy has been steadily rising worldwide over the past decade [10,11], emerging as one of the top 10 threats to global health listed by the WHO in 2019.

Community engagement is a process that involves engaging and motivating diverse partners to collaborate in harnessing community potential and enhancing community health [12,13]. It first gained prominence in the public health sphere with the Declaration of Alma-Ata and has since become increasingly prominent, particularly with the introduction of the new Sustainable Development Goals [14]. The WHO defines community engagement as “a process of developing relationship which enables stakeholders work together to address health issues” [15]. The United Nations Children’s Fund (UNICEF) defines community engagement as “an action for working with community stakeholders to improve community health” [13]. The definition of community engagement often intersects, competes with, and contradicts definitions of other terms such as community participation and community involvement, among others. Despite the extensive literature on community engagement, there is a lack of comprehensive guidelines to clarify the content and scope of community engagement, including what constitutes community engagement and the extent of its involvement. The levels of community engagement are structured along a continuum that spans from informing and consulting to involving, collaborating, and empowering [16,17]. The elements of community engagement manifest across a spectrum of initiatives, encompassing participant recruitment, intervention development, intervention implementation, and data collection [18,19]. Community engagement is characterized as a dynamic process rather than a singular intervention, operating within diverse contexts to address various issues through multiple mechanisms involving different actors.

A meta-analysis, incorporating 131 individual studies, supported the positive impact of community engagement on health and psychosocial outcomes for disadvantaged groups across various conditions [20]. It plays a prominent role in global immunization strategies, as it has the capacity to alleviate vaccination hesitancy and enhance vaccination confidence. A systematic review, which included 14 studies, examined the effectiveness of community interventions on HPV vaccine coverage. Of these, 12 studies reported that community interventions led to an increase in the uptake of the HPV vaccine [21]. Another review, spanning across 19 countries, assessed studies indicating that community engagement enhanced the timeliness and coverage of routine childhood immunization vaccines [22]. Despite robust evidence supporting the role of community engagement in promoting health within diverse populations, the evidence for community engagement specifically in vaccination promotion remains fragmented. Thus, we conducted a systematic review and meta-analysis to investigate the effectiveness of various aspects and levels of community engagement in enhancing vaccination rates.

## Methods

### Overview

This study was conducted following the guidelines outlined in the Cochrane Handbook for Systematic Reviews of Interventions [23], and the results were reported following the PRISMA (Preferred Reporting Items for Systematic Reviews and Meta-Analyses) guidelines [24]. The review protocol was registered in the PROSPERO database (CRD42022339081). Two reviewers (ML and YJX) conducted the literature search, study selection, quality assessment, and data extraction independently. Any discrepancies were resolved through discussion, and a third reviewer (LY) was consulted for arbitration when necessary.

### Ethics Approval

This review paper was a secondary analysis of existing data from original studies published before, rather than a direct collection of new data, and thus, does not require ethical approval.

### Search Strategies

A comprehensive and exhaustive literature search was conducted across 4 English databases (PubMed, Embase, Web of Science, and Cochrane Library), as well as 2 Chinese databases (CNKI and Wan Fang).

The search strategy involved combining terms related to “community engagement” and “vaccination” using specific vocabulary terms (MeSH and Emtree) and their corresponding free-text terms [25,26]. These terms were identified based on key publications in relevant fields, and the search strategy was adjusted to suit each database. Boolean operators, specifically “OR” between terms and “AND” between concepts, were used to combine search terms effectively.

No restrictions were placed on language, study design, country of origin, or publication date. Studies were searched in the selected databases from their inception to April 30, 2023. The initial literature searches were performed in June 2022, with an updated search conducted in April 2023. In addition, the reference lists of relevant articles and previous reviews were manually reviewed to identify any additional relevant studies. The ProQuest Dissertations & Theses Database was consulted to identify unpublished dissertations and theses. Furthermore, Google and Google Scholar were searched to identify gray literature for potential inclusion. Clinical trial registries, including ClinicalTrials.gov and the WHO International Clinical Trials Registry, were also searched to identify trials with outcomes that had not yet been published.

Details of the full search strategy for each database are listed in Table S1 in Multimedia Appendix 1.

### Selection Criteria

The inclusion and exclusion criteria were established based on the participants, interventions, comparisons, outcomes, and study design (PICOS) strategy [27]. Initially, these criteria were applied to titles and abstracts, and subsequently to full-text articles, to determine their final inclusion status (Table 1).

All records retrieved from the literature search were imported into the bibliographic database EndNote (Clarivate), which was used to manage records and eliminate duplicates. Two reviewers (ML and XL) independently screened the records based on the eligibility criteria. Any discrepancies between the 2 reviewers were resolved through discussion, and a third reviewer (YJX) was consulted if consensus could not be reached. The search terms and selection criteria were designed to provide inclusive flexibility and discretion, considering the various permutations of community engagement.

**Table 1:** Inclusion and exclusion criteria for literature.

Strategy	Inclusion criteria	Exclusion criteria
Population (P)	All age groups	No restrictions
Intervention (I)	Community engagement was required to meet 2 compulsory criteria [28,29]: (1) identify community partners in research and (2) engage community partners in intervention. Partner engagement was required to meet 4 optional criteria [28,29]: (1) participant recruitment, (2) intervention development, (3) intervention implementation, and (4) data collection.	Inability to identify community partners or failure to engage community partners.
Comparison (C)	Blank control, active control, and any other intervention	No restrictions
Outcome (O)	Vaccine rates that involved full immunization, partial immunization, and up-to-date immunization [30-32].	No data on vaccine rates
Study design (S)	Experimental designs that included randomized controlled trials, quasi-randomized controlled trials, non–randomized controlled trials, or controlled pre-post studies.	Descriptive or conceptual studies

### Data Extraction

A data extraction form was developed and piloted on 6 randomly selected sample studies to establish consensus on the data abstraction procedures. Subsequently, 2 independent investigators (ML and XL) extracted information including the first author, publication year, study design, country, participant number, intervention details, control condition, vaccine rates, and effect size of the intervention, where reported. In cases where a study provided data for both vaccine series initiation and completion, only the latter was included in the summary table. If a study evaluated multiple vaccine types and reported a combined vaccination rate, that result was selected; otherwise data for the primary vaccine under focus were presented. In instances where a study reported incomplete data, the authors were contacted via email to obtain the required information.

### Assessment of the Risk of Bias

The revised Cochrane Tool for Risk of Bias 2.0 (RoB2) was used to assess the risk of bias in randomized controlled trials (RCTs) [33]. For nonrandomized trials and controlled pre-post studies, the Risk of Bias in Non-randomized Studies-of Interventions (ROBINS-I) tool was used to evaluate the risk of bias [34].

Each study was assessed and categorized as having low, moderate, or high risk of bias for each domain. Studies with low risk in 3 or more domains and moderate risk in any remaining domain(s) were classified as having an overall low risk of bias. Studies with moderate risk in 3 or more domains and low or unclear risk in any remaining domain(s) were classified as having an overall moderate risk of bias. Studies with high risk in 3 or more domains and moderate risk in any remaining domain(s) were classified as having an overall high risk of bias. Studies with moderate risk in 3 or more domains and high risk in any remaining domain(s) were also classified as having an overall high risk of bias.

### Data Synthesis

Descriptive statistics were used to summarize the key variables of the included studies. Meta-analysis was conducted using Stata version 15.1 (StataCorp LLC) to investigate the effectiveness of community engagement in promoting vaccination.

Vaccination rates were computed as the proportion of vaccinated individuals to the total targeted population. Effect sizes were represented as the rate difference (RD) of vaccination rates, along with 95% CIs [35,36]. Random effects models were used to calculate pooled effect sizes, considering the expected heterogeneity among studies. Standard errors were adjusted for clustering effects when trials used a cluster randomized controlled design.

Forest plots were used to display individual and pooled vaccination rates. Heterogeneity was assessed using the Cochrane *Q* test (*P*_CQ_<.10) and the *I*^2^ statistics. Subgroup analyses were conducted based on age groups, vaccine types, and immunization. A meta-regression analysis was performed to explore the effects of study design or quality on the pooled effect size [37,38]. Sensitivity analysis using a single-study knockout approach was performed to determine the contribution of each study to the pooled effect size. Publication bias was evaluated through visual inspection of the funnel plot, and the asymmetry of the funnel plot was further assessed using the Egger test [39]. The Egger tests required a minimum of 10 publications to examine the association between SE and effect size in the funnel plot [37]. We classified the evidence quality into different levels according to the recommendations from van Tulder et al [40].

## Results

### Study Identification and Selection

**Figure 1:**
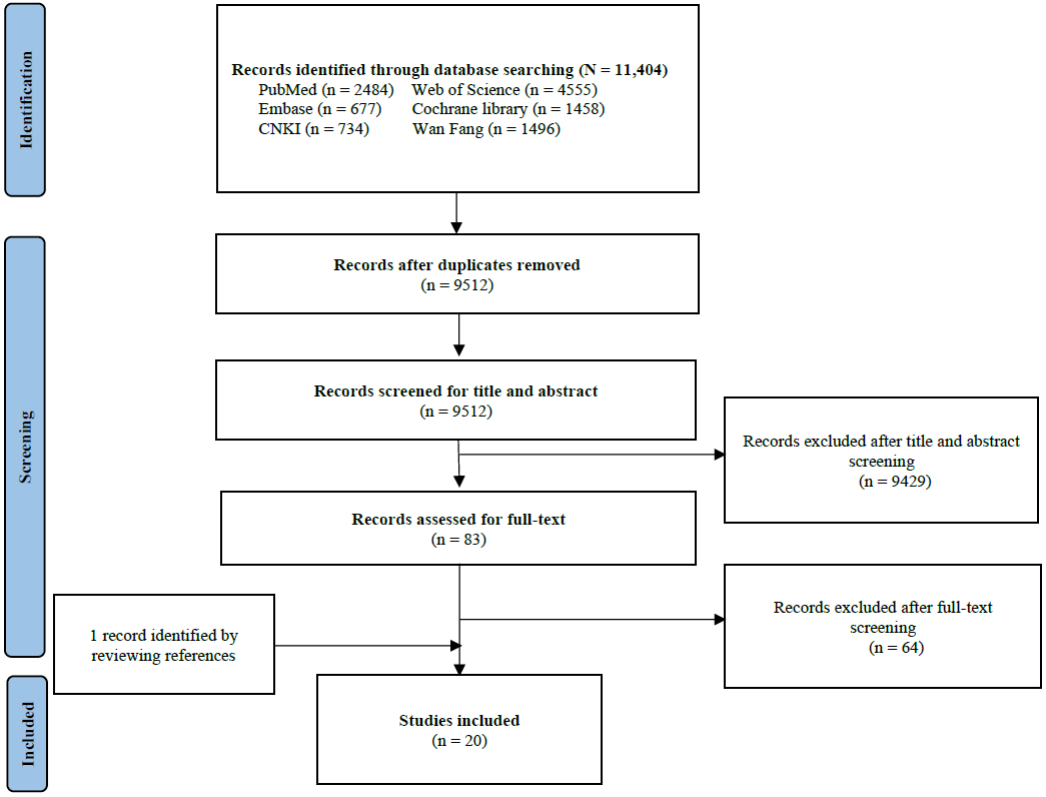
The flowchart of study selection. Community engagement is a process that involves engaging and motivating diverse partners to collaborate in harnessing community potential and enhancing community health.

The flowchart depicting the study selection process is presented in Figure 1. The literature search was conducted across 6 electronic databases from July 5, 2022, to July 12, 2022, yielding a total of 11,404 records. After removing duplicates, 9512 articles remained. Following the preliminary review of titles and abstracts, 83 articles were retained for full-text assessment. Subsequently, after reviewing the full texts, the final selection of 19 eligible articles was made. An additional article was identified through a manual search of reference lists. Therefore, a total of 20 eligible articles published in English were identified that met all inclusion criteria.

### Characteristics of the Included Studies

The characteristics of the included studies are summarized in Table S2 in Multimedia Appendix 1. This review did not restrict the timeframe of the literature search to provide a broad temporal perspective. The included studies were published between 2006 and 2021, with the majority (n=8) in the last 5 years. These studies were conducted in various countries, with the highest number (n=13) conducted in the United States [41-53], followed by Nigeria (n=3) [54-56] and Peru (n=2) [57,58], and 1 each in Pakistan [29] and India [59]. The studies used various designs, with over one-half (n=12) adopting single-group pre-post study designs [41-44,46-48,53,54,56-58], while the rest used cluster RCTs (n=5) [29,45,52,55,59] and non-RCTs (n=3) [49-51]. The studies recruited participants across all age groups, spanning from children (n=8) [29,43,44,46,54-56,59], to adolescents (n=7) [41,49-52,57,58], and to adults (n=5) [42,45,47,48,53]. The sample sizes of pre-post studies ranged from 30 to 12,103, with a median of 323, while the sample sizes of RCTs ranged from 337 to 2598, with a median of 349. These included studies targeted multiple vaccines, with 8 studies focusing on children’s immunization [29,43,44,46,54-56,59], 8 studies on HPV vaccine [41,47,49-52,57,58], 3 studies on hepatitis B virus (HBV) vaccine [42,45,53], and 1 study on COVID-19 vaccine [48]. Vaccination coverage was calculated using either individual-reported or officially recorded data.

### Conceptualization of Community Engagement

Community engagement does not neatly fit into predefined typologies, as it encompasses a variety of contexts, extents, and outcomes [60,61]. To address this complexity, a conceptual framework of community engagement was developed. This framework aims to delineate the different contents and extent of community engagement, drawing from the WHO definition of community engagement [62] and the utilitarian perspective of community engagement [63]. The contents of community engagement were delineated into 4 main categories: participant recruitment, intervention development, intervention implementation, and data collection. The extents of community engagement were categorized as low, moderate, and high [64]. Specifically, a low extent of community engagement indicated that studies fulfilled 1 or 2 contents of community engagement; a moderate extent of community engagement indicated that studies fulfilled 3 contents of community engagement; and a high extent of community engagement indicated that studies fulfilled all 4 contents of community engagement [64].

Most studies incorporated 2 engagement contents, with the majority engaged in intervention implementation (19/20, 95%) [29,41-46,48-59] and intervention development (13/20, 65%) [41-43,45-50,52-54,56], followed by participant recruitment (12/20,60%) [41,43-49,51,56-58] and outcome evaluation (11/20, 55%) [29,42-44,46,48,51,53-55,57] (Table 2). Furthermore, most studies fell into the moderate engagement extent category (n=10) [41,42,44,45,49,51,53,54,56,57], followed by low engagement extent (n=7) [29,47,50,52,55,58,59] and high engagement extent (n=3) [43,46,48] (Table 2).

**Table 2:** The contents and extent of community engagement in included studies.

Study	Participant recruitment (n=12)	Intervention development (n=13)	Intervention implementation (n=19)	Data collection (n=11)	The number of community engagement content	The extent of community engagement
Bailey et al [53]		✓	✓	✓	3	Moderate
Ma et al [45]	✓	✓	✓		3	Moderate
Weir et al [42]		✓	✓	✓	3	Moderate
Levinson et al [57]	✓		✓	✓	3	Moderate
Abuelo et al [58]	✓		✓		2	Low
Parra-Medina et al [51]	✓		✓	✓	3	Moderate
Lee et al [47]	✓	✓			2	Low
Paskett et al [52]		✓	✓		2	Low
Sanderson et al [50]		✓	✓		2	Low
Lennon et al [41]	✓	✓	✓		3	Moderate
Ma et al [49]	✓	✓	✓		3	Moderate
Findley et al [43]	✓	✓	✓	✓	4	High
Willis et al [46]	✓	✓	✓	✓	4	High
More et al [59]			✓		1	Low
Habib et al [29]			✓	✓	2	Low
Bawa et al [54]		✓	✓	✓	3	Moderate
Oyo-Ita et al [55]			✓	✓	2	Low
Akwataghibe et al [56]	✓	✓	✓		3	Moderate
Suryadevara et al [44]	✓		✓	✓	3	Moderate
Marquez et al [48]	✓	✓	✓	✓	4	High

Community engagement in these studies took various forms of intervention strategies, including social marketing campaigns, community mobilization, health education and discussions, health service support, and follow-up and reminders. These interventions were often combined into intervention packages, which included combinations such as health education and discussion with follow-up and reminders, health education and discussion with health service support, health education and discussion with health service support and follow-up reminders, social marketing campaigns and community mobilization with health service support and follow-up reminders, and social marketing campaigns and community mobilization with health education and discussion as well as follow-up reminders.

Community engagement varied in geographical coverage, ranging from localized sites in 1 village or city to broader areas encompassing 1 district or more. However, many interventions failed to consider implementation constraints and practicalities on the ground, which in turn limited the fidelity of community engagement and the efficient utilization of community resources.

### Risk-of-Bias Assessment

These studies exhibited variable quality across different study designs, with none meeting all the elements of a good quality design. Individual domain ratings and overall bias risk ratings for each study are presented in Tables S3 and S4 in Multimedia Appendix 1.

Two cluster RCT studies [29,59] were identified as having a low risk of bias, 1 [55] as a moderate risk of bias, and 2 [45,52] as a high risk of bias. This variability in the risk of bias may result from incorrect randomization procedures, deviations in intervention implementation, and incomplete outcome reporting. In addition, 6 quasi-experimental studies [44,48,49,53,54,57] were rated as having a low risk of bias, 4 [42,46,47,56] were rated as having a moderate risk of bias, and 5 [41,43,50,51,58] were rated as having a high risk of bias. The sources of bias in these studies may include confounding effects, missing outcome data, and selective reporting of results.

### Overall Meta-Analysis of Community Engagement on Vaccination Rates

**Figure 2:**
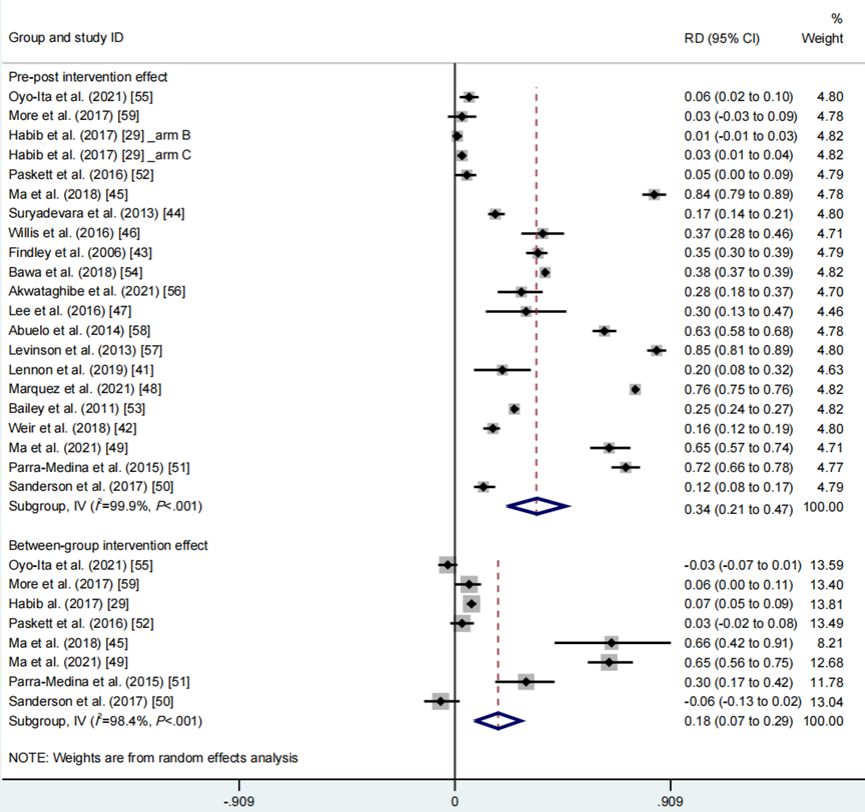
Meta-analysis of the effects of overall community engagement on vaccination rates. See also [29,41-59]. RD: rate difference.

The pooled meta-analysis incorporated usable data from 21 intervention groups across 20 studies. The random effects meta-analysis of pre-post intervention effects revealed a moderate positive effect size of community engagement on promoting vaccination rates (RD 0.34, 95% CI 0.21-0.47, *I*^2^=99.9%, *P*_CQ_<.001; Figure 2, see also [29,41-59]). Similarly, the random effects meta-analysis of between-group intervention effects showed a small positive effect size of community engagement on promoting vaccination rates (RD 0.18, 95% CI 0.07-0.29, *I*^2^=98.4%, *P*_CQ_<.001; Figure 2).

### Meta-Analysis of Community Engagement Contents and Extent on Vaccination Rates

**Figure 3:**
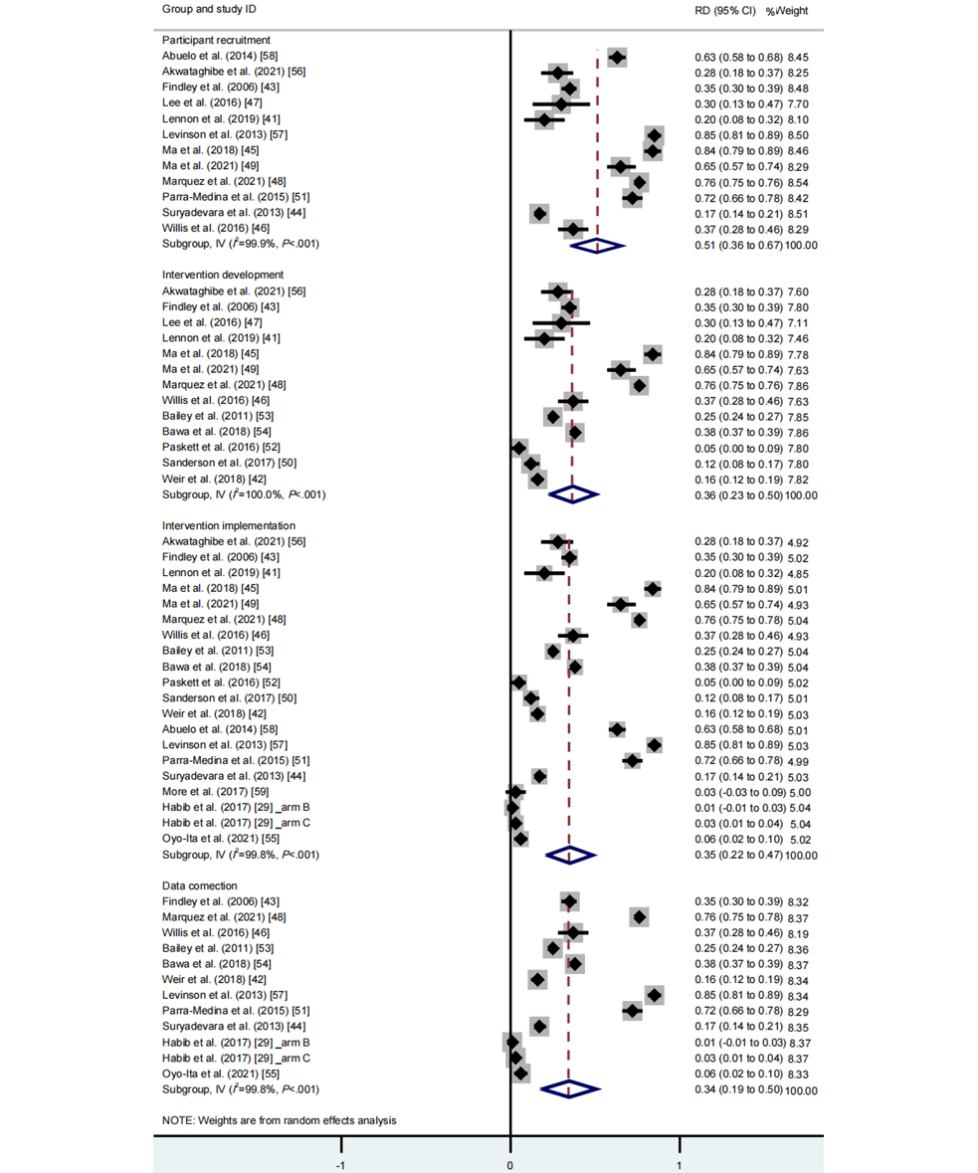
Meta-analysis of the effects of different contents of community engagement on vaccination rates. See also [29,41-59]. RD: rate difference.

Regarding the contents of community engagement, the random effects meta-analysis revealed that participant recruitment yielded the largest effect size (RD 0.51, 95% CI 0.36-0.67, *I*^2^=99.9%, *P*_CQ_<.001), followed by intervention development (RD 0.36, 95% CI 0.23-0.50, *I*^2^=100.0%, *P*_CQ_<.001), intervention implementation (RD 0.35, 95% CI 0.22-0.47, *I*^2^=99.8%, *P*_CQ_<.001), and data collection (RD 0.34, 95% CI 0.19-0.50, *I*^2^=99.8%, *P*_CQ_<.001; Figure 3, see also [29,41-59]).

**Figure 4:**
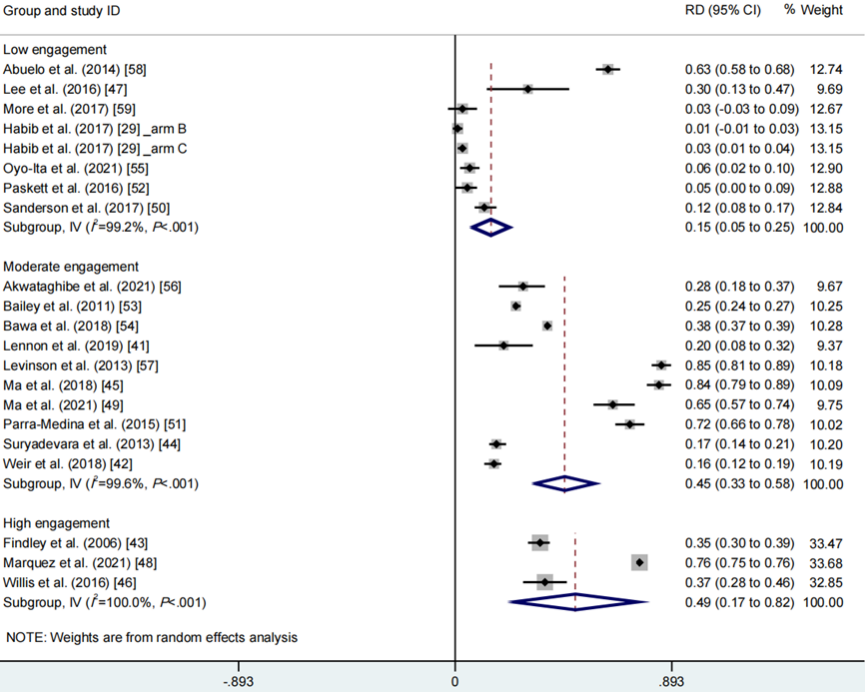
Meta-analysis of the effects of different extents of community engagement on vaccination rates. See also [29,41-59]. RD: rate difference.

With regard to the extent of community engagement, the random effects meta-analysis found that high community engagement extent yielded the largest effect size (RD 0.49, 95% CI 0.17-0.82, *I*^2^=100.0%, *P*_CQ_<.001), followed by moderate community engagement extent (RD 0.45, 95% CI 0.33-0.58, *I*^2^=99.6%, *P*_CQ_<.001) and low community engagement extent (RD 0.15, 95% CI 0.05-0.25, *I*^2^=99.2%, *P*_CQ_<.001; Figure 4, see also [29,41-59]).

### Meta-Analysis of Intervention Strategies on Vaccination Rates

**Figure 5:**
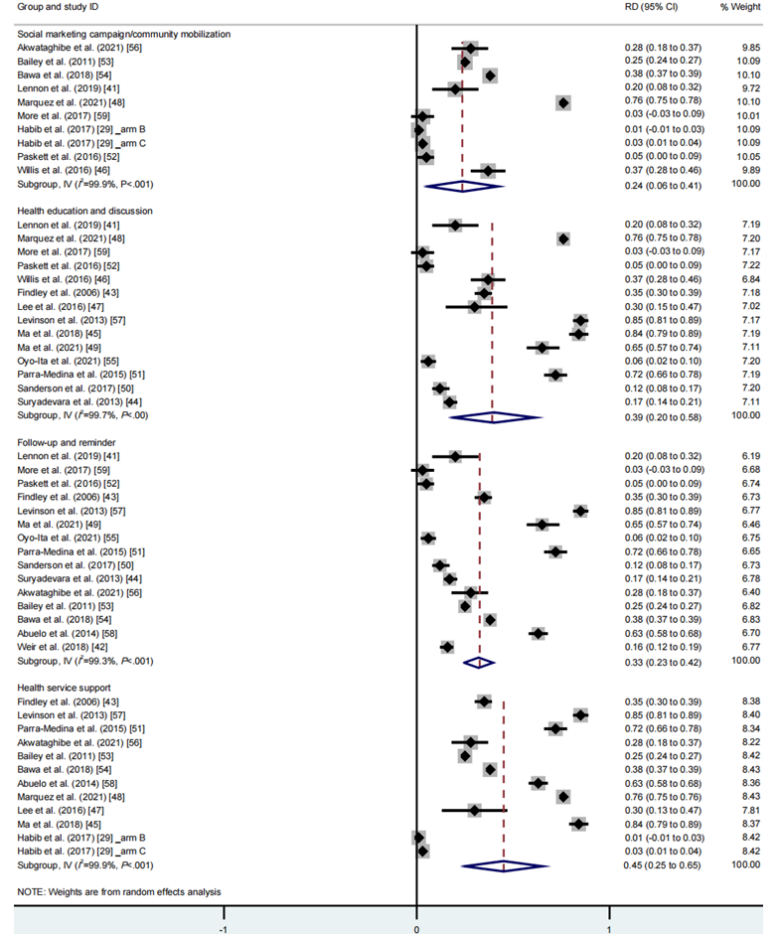
Meta-analysis of the effects of a single component of intervention strategy on vaccination rates. See also [29,41-59]. RD: rate difference.

With regard to single types of intervention strategies, the meta-analysis of 4 intervention strategies found that “health service support” yielded the largest effect sizes (RD 0.45, 95% CI 0.25-0.65, *I*^2^=99.9%, *P*_CQ_<.001), followed by “health education and discussion” (RD 0.39, 95% CI 0.20-0.58, *I*^2^=99.7%, *P*_CQ_<.001), “follow-up and reminder” (RD 0.33, 95% CI 0.23-0.42, *I*^2^=99.3%, *P*_CQ_<.001), and “social marketing campaigns and community mobilization” (RD 0.24, 95% CI 0.06-0.41, *I*^2^=99.9%, *P*_CQ_<.001; Figure 5, see also [29,41-59]).

**Figure 6:**
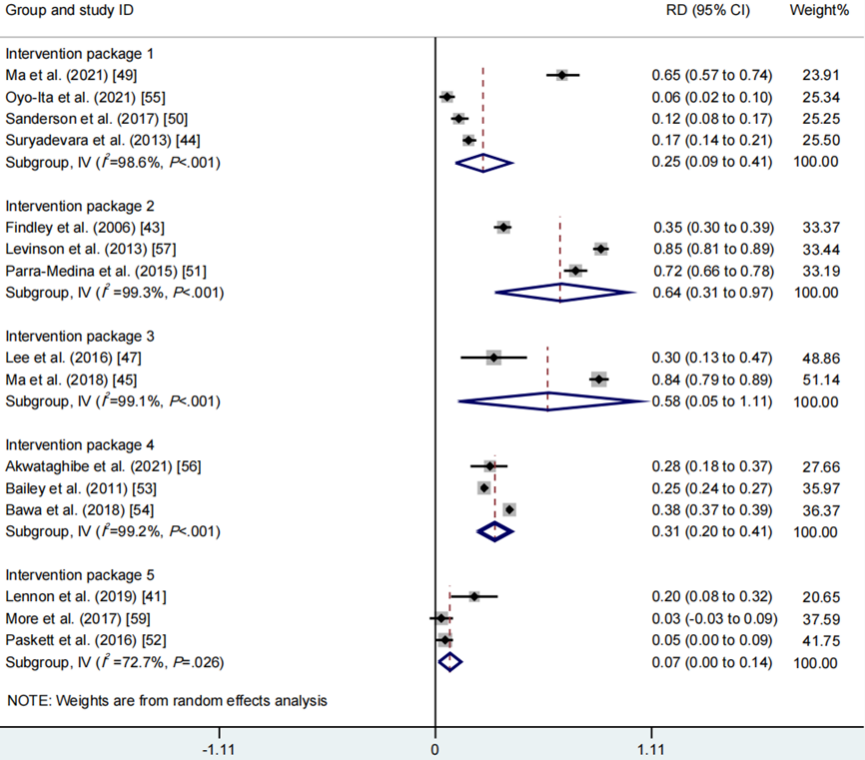
Meta-analysis of the effects of multicomponents of intervention strategy on vaccination rates. RD: rate difference.

With regard to combined types of intervention strategies, the meta-analysis of 5 intervention strategy packages found that intervention package 2 yielded the largest increase in vaccination rates (RD 0.64, 95% CI 0.31-0.97, *I*^2^=99.3%, *P*_CQ_<.001), followed by intervention package 3 (RD 0.58, 95% CI 0.05-1.11, *I*^2^=99.1%, *P*_CQ_<.001), intervention package 4 (RD 0.31, 95% CI 0.20-0.41, *I*^2^=99.2%, *P*_CQ_<.001), and intervention package 1 (RD 0.25, 95% CI 0.09-0.41, *I*^2^=98.6%, *P*_CQ_<.001). However, intervention package 5 had no statistically significant impact on vaccination rates (RD 0.07, 95% CI 0.00-0.14, *I*^2^=72.7%, *P*_CQ_=.03; Figure 6, see also [41,43-45,47,49-57,59]). Data from 5 studies were not synthesized because of high heterogeneity in their intervention strategies [29,42,46,48,58].

### Subgroup Analyses of Age Groups, Vaccine Types, and Immunization Definitions on Vaccination Rates

Subgroup analyses revealed that adults (RD 0.50, 95% CI 0.16-0.85, *I*^2^=100.0%, *P*_CQ_<.001) exhibited a larger effect size compared with adolescents (RD 0.44, 95% CI 0.18-0.70, *I*^2^=99.3%, *P*_CQ_<.001) or children (RD 0.18, 95% CI 0.04-0.33, *I*^2^=99.7%, *P*<.001; Figure S1 in Multimedia Appendix 1). HPV vaccination (RD 0.44, 95% CI 0.18-0.70, *I*^2^=99.3%, *P*_CQ_<.001) exhibited a larger effect size compared with HBV vaccination (RD 0.42, 95% CI 0.12-0.72, *I*^2^=99.8%, *P*_CQ_<.001) or children immunization (RD 0.18, 95% CI 0.04-0.33, *I*^2^=99.7%, *P*_CQ_<.001; Figure S2 in Multimedia Appendix 1). Full immunization (RD 0.41, 95% CI 0.30-0.53, *I*^2^=99.5%, *P*_CQ_<.001) exhibited a larger effect size compared with partial immunization (RD 0.20, 95% CI 0.08-0.33, *I*^2^=93.3%, *P*_CQ_<.001). However, no significant increase was found in the vaccine rate of up-to-date immunization (RD 0.25, 95% CI –0.10 to 0.60, *I*^2^=100.0%, *P*_CQ_<.001; Figure S3 in Multimedia Appendix 1).

### Sensitivity, Meta-Regression, Publication Bias, and Evidence Level

Sensitivity analysis showed that no significant changes were observed in the effect size of the pre-post intervention effect analysis (Figure S4 in Multimedia Appendix 1). However, the pooled effect size decreased dramatically when eliminating the study of Ma et al [45] in the between-group intervention effect analysis (RD 0.08, 95% CI 0.02-0.20; Figure S5 in Multimedia Appendix 1). Meta-regression analyses did not show any association between effect size and study design or study quality for the pre-post intervention effect analysis (*P*=.16 or *P*=.65; Figure S6 in Multimedia Appendix 1). As evidenced by the funnel plot and Egger test, no discernible signs of publication bias were detected either in the pre-post or in the between-group intervention effect analyses (*P*=.25; Figures S7 and S8 in Multimedia Appendix 1). According to recommendations from van Tulder et al [40], evidence quality in our meta-analysis was graded as moderate in both the pre-post and between-group intervention effect analyses (Table S5 in Multimedia Appendix 1).

## Discussion

### Principal Findings

Community engagement drives interventions operated in a bottom-up manner rather than the traditional top-down approach. This approach supports stakeholders coming together to achieve global vaccination coverage goals from childhood to adulthood.

This study reported that community engagement strategies resulted in a 34% increase in vaccination rates through a pre-post intervention effect analysis and an 18% increase in vaccination rates through a between-group intervention effect analysis. The random effects meta-analyses indicated that participant recruitment exhibited the largest effect size, followed by intervention development, intervention implementation, and data collection. Consistent with previous evidence [64], intervention implementation constituted the primary engagement approach of most included studies and yielded a moderate effect size, while participant recruitment represented the engagement approach of over half of the included studies and yielded the largest effect size. Community partners who possess the knowledge and skills to effectively approach the target population and actively engage in participant recruitment hold the most potential to achieve relatively high recruitment and retention rates for participants. This meta-analysis found that the effect size increased with the extent of community engagement, with the highest community engagement extent exhibiting the largest effect size. Similar to previous evidence [65], a higher extent of community engagement resulted in greater vaccination promotion. Previous systematic reviews, which classify community engagement into different levels from low to high, also reported positive correlations between community engagement extents and intervention effects [65]. Regarding intervention strategies, the meta-analyses showed that health service support yielded the largest effect size, followed by health education and discussion, follow-up and reminder, and social marketing campaigns and community mobilization. Similar to previous studies, health service support increased routine childhood vaccine coverage [66]; health education and discussion increased HPV vaccine coverage among adolescents [21,67] and influenza vaccine coverage among older adults [68]; follow-up and reminder increased HBV vaccine coverage among adults [69]; and social marketing campaigns and community mobilization increased routine childhood vaccine coverage [70]. Health service support, whether used alone or in combination with other strategies, demonstrated effectiveness for vaccination promotion. Consistent with our analysis, previous studies have supported the effectiveness of health service support in the form of free vaccination, vaccination outreach or mobile clinic vaccination [66,71], and flexible vaccination schedules [72]. Intervention packages that combined health service support with the other 3 strategies resulted in a significant boost in vaccine rates. The intervention packages with adaptability and flexibility, which incorporated diverse intervention strategies, could effectively meet the needs of the community population and maximize intervention benefits.

Meta-analyses across a broad range of topics, populations, and interventions often encounter a disjunction between considerable heterogeneity arising from broad questions and the limited statistical methods available for variance analysis. The limited number of included studies precluded the performance of subgroup analyses and meta-regressions to fully address the sources of heterogeneity. The development of a conceptual framework provided homogeneity at the theoretical level despite the unavoidable nature of situational heterogeneity.

The geographic coverage of the included studies spanned across 5 countries, with most studies located in the United States, which could reflect a type of publication bias along with the skewed nature of global health research. These included studies were published between 2006 and 2021, with the majority in the last 5 years, which could reflect increased academic enthusiasm and enhanced policy support in recent years. However, most studies failed to disclose the social characteristics of community participants, which highlights the reality of known social hierarchies within communities.

Many studies proposed operational definitions of community engagement, and some studies suggested empirical models to explain its connotation. However, few articles made references to definitions or frameworks, reflecting a lack of theoretical basis and critical perspective. The lack of common definitions, along with the absence of conceptual frameworks, has led to diversified procedures and contents of engagement across diverse contexts and practices. Despite the wide acceptance of community engagement in theory and practice, considerable challenges remain in identifying the best engagement approach and evaluating engagement effectiveness [73-75]. Community engagement shares similar spirits but varies in practices, as the extent of engagement spans a spectrum from minimal superficial involvement to fully collaborative participation. Operating community engagement is cost-intensive rather than cost-neutral, requiring labor, capital, and time to establish, develop, and sustain fruitful partnerships, thus posing challenges to its successful and sustainable implementation. These included studies failed to report any analysis of costs, which precluded conclusions about the economic case for community engagement. While studies support the value of community engagement, the evaluation of community engagement has largely focused on health outcomes and ignored economic information. Future studies should incorporate economic analysis to explore the potential cost-utility and cost-effectiveness of community engagement in real-world contexts. This will help close the research-practice gap and facilitate evidence-based policy making.

The inclusion of experimental designs allowed the identification of a clear link between community engagement and vaccination promotion. However, none of these included studies were located at the top level of the evidence hierarchy, which limited the direct contribution of community engagement to vaccination promotion. Future studies with more rigorous designs should be performed to draw more definitive linkages about which participant group benefits the most from which engagement type in what community context. Randomized trials followed allocation sequence methods to ensure between-group comparability, but most interventions differed from comparisons in more ways than just community engagement. The comparator for community engagement always involved a completely different multicomponent intervention rather than the conventional health promotion activity without community engagement. The lack of a pure comparator in most community engagement interventions could cloud the interpretation of this meta-analysis. Community engagement often operates in nonlinear pathways synergized between various components and multiple outcomes, thus complicating effect evaluation compared with simple dose-response relationships. Community engagement functions as a dynamic process rather than as a discrete intervention, implying that evaluation should fully account for intrinsic complexities rather than simply focusing on outcome indicators. The primary studies should conduct thorough process evaluations to incorporate a spectrum of outcome measures and complement qualitative evaluations to elucidate the active ingredients of community engagement and the potential unintended effects of community engagement.

The effects of community engagement on vaccination promotion did not occur as a linear progression, but rather consisted of complex processes influenced by facilitators or challenges. These included studies identified individual- and community-level factors that facilitated or challenged community engagement in the context of vaccination promotion. At the individual level, the sense of confidence and ownership, along with the development of leadership skills and knowledge, facilitated community partners to engage with participatory processes. Conversely, the lack of interest and capacity, as well as the ambiguity of role and responsibility, challenged community partners to engage with participatory processes. At the community level, trust facilitated effective community engagement, while mistrust inhibited genuine community engagement. Further work should adopt a broader range of study designs that encompass both quantitative and qualitative methodologies to measure these intangible facilitators or challenges in the area of community engagement.

These included studies faced the challenge of measuring the level of community engagement, as engagement levels span a spectrum from more passive involvement to more active participation. This study proposed operationalized extents of community engagement beyond levels of community engagement from a pragmatic perspective. Further studies should be performed to develop tools or standards to measure and evaluate the levels of community engagement effectively.

As most studies narrowly defined community engagement as an intervention program imposed on the community, they framed the effectiveness of community engagement in terms of short-term individual-level outcomes [60] while neglecting multidimensional community-level outcomes. A narrow definition of community engagement, along with a restricted view of effectiveness, excludes a conceptually coherent and methodologically sound evaluation of community engagement [15]. Evaluating community engagement raises a unique set of challenges around conceptual, methodological, and practical aspects [76]. The interaction between the engagement strategy and the community system creates a degree of complexity beyond the detail of intervention implementation [77]. This complexity grows in concert with the delivery of the engagement strategy, which may, in some instances, reshape the intervention and the community context [77]. Future work should focus on intervention theories, logic models, and outcome frameworks to clarify the relationship between community engagement and health outcomes.

Community engagement can function independently or in conjunction with other initiatives. However, when combined with other initiatives, it becomes challenging to isolate the specific contribution of community engagement to health outcomes [78]. On the other hand, some studies treated community engagement as a discrete intervention rather than a dynamic process. This oversight has resulted in a lack of alternative process evaluations to explore how community engagement contributes to vaccination promotion [79-81]. Despite the widespread use and recognized significance of community engagement [82], there are still gaps in measuring and evaluating its implementation. While there is a vast body of literature on community engagement spanning various disciplines, comprehensive guidelines and frameworks for community engagement are lacking. The adoption of consistent guidelines and frameworks can formalize the implementation and evaluation of community engagement efforts.

### Limitations

This study faces some challenges and limitations that warrant consideration and point toward future directions. The first challenge was the range of different definitions and terminology referring to engagement versus involvement and participation. The second challenge was the disjunction between the conceptual heterogeneity inherent in such broad questions and the limited statistical methods available to analyze variance. The third limitation was the possibility of study omission due to search deficiencies or publication bias, despite the extensive and rigorous literature search conducted.

### Conclusions

The findings of this meta-analysis support the effectiveness of community engagement in promoting vaccination, with variations observed in terms of the contents and extent of engagement. Experimental studies often involve differences between the intervention and comparison groups beyond just community engagement. Studies designed to specifically isolate community engagement as the only differing factor between the intervention and comparison groups are suggested, which allows for a clearer understanding of its added value in vaccination promotion. Comprehensive process evaluations and qualitative evaluations should be used, to provide insights into the active ingredients of community engagement and uncover any unintended effects it may have. A further scientific agenda on community engagement should focus on theory development, framework construction, and effectiveness evaluation. Future studies will benefit from the adoption of standard guidelines and frameworks to enable cross-study or cross-country comparisons of community engagement, promoting effective, sustainable, and appropriate community initiatives.

## Appendix

Multimedia Appendix 1 Literature search results, characteristics of included studies, risk-of-bias assessment, and subgroup and sensitivity analyses.

## Abbreviations

HBV: hepatitis B virus
HPV: human papillomavirus
PICOS: participants, interventions, comparisons, outcomes, and study design
PRISMA: Preferred Reporting Items for Systematic Reviews and Meta-Analyses
RCT: randomized controlled trial
RD: rate difference
ROBINS-I: Risk of Bias in Non-randomized Studies-of Interventions
UNICEF: United Nations Children’s Fund
WHO: World Health Organization

## Data Availability

All data generated or analyzed during this study are included in this published article and its supplementary information files.
