# Supplementary material for: Community Engagement in Vaccination Promotion: Systematic Review and Meta-Analysis
Source: JMIR Public Health Surveill. 2024 May 10;10:e49695. doi: 10.2196/49695 (PMC11127135; doi:10.2196/49695)
Supplement: Multimedia Appendix 1 [file publichealth_v10i1e49695_app1.docx]

**Appendix File**

Table S1 Literature search results in six electronic databases

Table S2 Characteristics of included studies

Table S3 Details of risk of bias assessment for 5 RCT studies

Table S4 Details of risk of bias assessment for12 pre-post studies and 3 on-RCT studies

Table S5. The levels of evidence strength

Figure S1 Subgroup analysis of the effects on vaccination rates by age groups (N= 20 studies)

Figure S2 Subgroup analysis of the effects on vaccination rates by vaccine types (N= 20 studies)

Figure S3 Subgroup analysis of the effects on vaccination rates by immunization definitions (N= 20 studies)

Figure S4 Sensitivity analysis for the effects of community engagement on vaccination rates in the

pre-post intervention effect analysis (N= 20 studies)

Figure S5 Sensitivity analysis for the effects of community engagement on vaccination rates in the intervention-control comparison effect analysis (N=8 studies)

Figure S6 Meta-regression analysis for the effects of community engagement on vaccination rates in the pre-post intervention effect analysis (N= 20 studies)

Figure S7 Funnel plot of publication bias in the effects of community engagement on vaccination rates in the pre-post intervention effect analysis (N= 20 studies)

Figure S8 Egger test of publication bias in the effects of community engagement on vaccination rates in the pre-post intervention effect analysis (N= 20 studies)

| **DATABASES** | **Results*** |
| --- | --- |
| **PubMed** | 2,484 |
| Search:  (((("Vaccines"[Mesh]) OR "Vaccination"[Mesh]) OR "Vaccination Coverage"[Mesh]) OR (((vaccin*[Title/Abstract]) OR (immuniz*[Title/Abstract]))  OR (immunis*[Title/Abstract]))) AND ((("Community-Based Participatory Research"[Mesh]) OR "Community Participation"[Mesh]) OR  (((((((((((((((((((((((((((((((((((((Community Based Participatory Research [Title/Abstract]) OR (Participatory Research, Community-Based [Title/Abstract])) OR (Consumer-Driven Community-Based Research [Title/Abstract])) OR (Community-Based Research, Consumer-Driven [Title/Abstract])) OR (Community-Based Research, Consumer-Driven [Title/Abstract])) OR (Consumer Driven Community Based Research [Title/Abstract])) OR (Consumer-Driven Community-Based Research [Title/Abstract])) OR (Research, Consumer-Driven Community-Based [Title/Abstract])) OR (Research, Consumer-Driven Community-Based [Title/Abstract])) OR (Participation, Community [Title/Abstract])) OR (Community Involvement [Title/Abstract])) OR (Community Involvements [Title/Abstract])) OR (Involvement, Community [Title/Abstract])) OR  (Involvements, Community [Title/Abstract])) OR (Consumer Participation [Title/Abstract])) OR (Participation, Consumer [Title/Abstract])) OR (Consumer Involvement [Title/Abstract])) OR (Consumer Involvements [Title/Abstract])) OR (Involvement, Consumer [Title/Abstract])) OR  (Public Participation [Title/Abstract])) OR (Participation, Public [Title/Abstract])) OR (Community Action [Title/Abstract])) OR (Action, Community [Title/Abstract])) OR (Actions, Community [Title/Abstract])) OR (Community Actions [Title/Abstract])) OR (participatory research [Title/Abstract])) OR (participatory action research [Title/Abstract])) OR (participatory approach [Title/Abstract])) OR (community engag*[Title/Abstract])) OR (community invovl*[Title/Abstract])) OR (community participat*[Title/Abstract])) OR (community stakeholder*[Title/Abstract])) OR (community partner*[Title/Abstract]))) OR (community-centered activit*[Title/Abstract])) OR (community-based collaborat*[Title/Abstract])) OR (academic community [Title/Abstract])))  **Other databases**   Embase | 677 |
|  Web of Science | 4,555 |
|  Cochrane Libary | 1,458 |
|  CNKI | 734 |
|  Wan Fang | 1,496 |

**Table S1 Literature search results in six electronic databases**

**TOTAL SEARCH RESULT 11,404**

**Table S2 Characteristics of included studies**

| **Author (year)** | **Country** | **Study design** | **Participants** | **Intervention** | **Comparation** | **Vaccine types** | **Vaccination outcomes** |
| --- | --- | --- | --- | --- | --- | --- | --- |
| Bailey et al. (2011) [53] | USA | Uncontrolled pre-post study | Asian/Pacific islanders (N=1962) | SMCs/CMs  Follow ups and reminders  Health service support | NA | HBV | Full immunization (three doses)  (1) Preintervention: 0%  (2) Postintervention: 25.5% (500/1962) |
| Ma et al. (2018) [45] | USA | Cluster RCT | Korean American aged 18 years or above (N=349) | Health education and discussion  Health service support | Health education about general cancer and health issues | HBV | Full immunization (three doses)  (1) Control group: 17.6% (3/17)  (2) Intervention group: 84% (279/332) |
| Weir et al. (2018) [42] | USA | Uncontrolled pre-post study | People aged 18-70 years (N=1914) | Follow ups and reminders | NA | HBV | Partial immunization (at least 1 dose)  (1) Preintervention: 11.7% (111/929)  (2) Postintervention: 27.9% (275/ 985) |
| Levinson et al. (2013) [57] | Peru | Uncontrolled pre-post study | Caregivers (N= 323)  Girls aged 10-13 years (N=352) | Health education and discussion Follow ups and reminders  Health service support | NA | HPV | Full immunization (three doses)  (1) Preintervention: 0%  (2) Postintervention: 85.2% (300/352) |
| Abuelo et al. (2014) [58] | Peru | Uncontrolled pre-post study | Caregivers (N= 320)  Girls aged 10-13 years (N=318) | Follow ups and reminders  Health service support | NA | HPV | Full immunization (three doses)  (1) Preintervention: 0%  (2) Postintervention: 62.9% (200/318) |
| Parra-Medina et al. (2015)[51] | USA | Non-RCT | Hispanic women (N=372)  Daughter aged 11-17 years (N=372) | Health education and discussion Follow ups and reminders  Health service support | HPV vaccine educational brochures | HPV | Full immunization (three doses)  (1) Control group: 42.5%  (2) Intervention group: 72.2% |
| Lee et al. (2016) [47] | USA | Uncontrolled pre-post study | Women aged 21-29 years（N=30） | Health education and discussion  Health service support | NA | HPV | Partial immunization (1 dose)  (1) Preintervention: 0% (0/30)  (2) Postintervention: 30% (9/30) |
| Paskett et al. (2016) [52] | USA | Cluster RCT | Caregivers (N= 337)  Adolescents aged 9-17 years  (N= 337) | SMCs/CMs  Health education and discussion  Follow ups and reminders | Health education about influenza and vaccine | HPV | Full immunization (three doses)  (1) Control group: 1.6% (2/124)  (2) Intervention group:4.6% (6/130) |
| Sanderson et al. (2017) [50] | USA | Non-RCT | Mothers (N=305)  Adolescent aged 9-18 years  (N= 408) | Health education and discussion Follow ups and reminders | Usual community care | HPV | Full immunization (three doses or more)  (1) Control group: 18.0 % (30/167)  (2) Intervention group: 12.4 % (24/194) |
| Lennon et al. (2019) [41] | USA | Uncontrolled pre-post study | Parents/ caregivers (N=118)  Adolescents aged 13-17 years (N=118) | SMCs/CMs  Health education and discussion  Follow ups and reminders | NA | HPV | UTD immunization  (1) Preintervention: 25.4% (30/118)  (2) Postintervention: 45.8% (54/ 118) |
| ﻿Ma et al. (2021) [49] | USA | Non-RCT | Chinese American caregivers (N=180)  Adolescent aged 11-18 years (N=180) | Health education and discussion Follow ups and reminders | General health education | HPV | Full immunization (three doses)  (1) Control group: 0% (0/70)  (2) Intervention group: 65.5% (72/110) |
| Findley et al. (2006) [43] | USA | Uncontrolled pre-post study | Caregivers (N= no information)  Children aged 19-35 months (N=1502) | Health education and discussion Follow ups and reminders  Health service support | NA | Immunization antigen series 4:3:1:3:3 | UTD immunization  (1) Preintervention: 46%  (2) Postintervention: 80.5% |
| Willis et al. (2016) [46] | USA | Uncontrolled pre-post study | Caregivers (N=no information)  Children aged 9-35 months (N=189) | SMCs/CMs  Health education and discussion | NA | ﻿Immunization antigen series 4:3:1:3:3:1 | UTD immunization  (1) Preintervention: 45%  (2) Postintervention: 82% |
| More et al. (2017) [60] | India | Cluster RCT | Caregivers (N= no information)  Children aged 12-23 months (N=2251) | SMCs/CMs  Health education and discussion  Follow ups and reminders | Routine immunization | BCG  DPT3  Polio  HBV3  Measles | Full immunization  (1) Control group: 61.9% (708/1143)  (2) Intervention group: 67.8% (751/1108) |
| Habib et al. (2017) [59] | Pakistan | Cluster RCT | Caregivers (N= no information)  Children aged 1 month to 5 years (N= 87984) | SMCs/CMs  Health service support | Routine immunization | OPV  EPI | (1) OPV  Control group (arm A): 75%  Intervention group (arm B): 82%  Intervention group (arm C): 84%  (2) EPI  Control group (arm A): 25%  Intervention group (arm B): 32%  Intervention group (arm C): 34% |
| Bawa et al. (2018) [54] | Nigeria | ﻿ Uncontrolled pre-post study | Mothers (N= no information)  Children aged under 1years (N=222879) | SMCs/CMs  Follow ups and reminders  Health service support | NA | OPV3  OPV  Penta 3 | (1) OPV3 (children under 1year)  Preintervention: 23%  Postintervention: 61%  (2) OPV (children aged 1-5 years)  Preintervention: 60%  Postintervention: 90%  (3) Penta 3 (children under 1year)  Preintervention: 22%  Postintervention: 55% |
| Oyo-Ita et al. (2021) [55] | Nigeria | Cluster RCT | Caregivers (N= 2598)  Children aged 0-23 months (N=2598) | Health education and discussion Follow ups and reminders | Routine immunization | BCG  OPV  Penta 3  PCV  Measles  Yellow fever | UTD immunization  (1) Control group: 54.7% (697/1274)  (2) Intervention group: 51.8% (661/1276) |
| Akwataghibe et al. (2021) [56] | Nigeria | Uncontrolled pre-post study | Caregivers (N=282)  Children aged 9 - 59 months  (N= 340) | SMCs/CMs  Follow ups and reminders  Health service support | NA | DPT3  Penta3  Measles  Yellow fever | Full immunization  (1) Preintervention: 19.8% (34/172)  (2) Postintervention: 47.6% (80/168) |
| Suryadevara et al. (2013) [44] | USA | Uncontrolled pre-post study | Caregivers (N= 630)  Children aged under 17 years (N=1531) | Health education and discussion, follow ups and vaccination reminders | NA | PCV-13  TIV  HPV Meningococcal | Full immunization  (1) Preintervention: 28.2% (416/477)  (2) Postintervention: 45.5 % (672/1477) |
| Marquez et al. (2021) [48] | USA | Uncontrolled pre-post study | People aged over 16 years (N=12103) | SMCs/CMs  Health education and discussion Health service support | NA | COVID-19 | UTD immunization (2 doses)  (1) Preintervention: 0% (0/12103)  (2) Postintervention: 75.6% (9152/12103) |

Abbreviation:

**NA**: not applicable **RCT**: randomized controlled trial **HBV**: hepatitis B virus vaccine **HPV**: human papillomavirus vaccine **UTD immunization**: up-to-date immunization **Immunization antigen series 4:3:1:3:3**: 4 diphtheria-tetanus-pertussis, 3 polio, 1 measles-mumps-rubella, 3 Haemophilus influenza b, 3 Hepatitis B **Immunization antigen series 4:3:1:3:3:1**: 4 diphtheria-tetanus-acellular pertussis; 3 polio; 1 measles-mumps-rubella; 3 hepatitis b; 3 Haemophilus influenzae b; 1 varicella **BCG**: Bacillus Calmette-Guerin vaccine **DPT3**: Diphtheria, Tetanus, Pertussis (three doses) **OPV3**: oral polio vaccine (three dose) **Routine EPI vaccines**: BCG, Penta3, measles vaccine **Penta 3**: Diphtheria, Tetanus, Pertussis, hepatitis b; Haemophilus influenzae b (three doses) **IPV**: inactivated polio vaccine **PCV13**: pneumococcal conjugate vaccine-13 vaccine **TIV**: trivalent influenza vaccine **MMR vaccine**: measles-mumps-rubella vaccine **SMCs/CMs**: social marketing campaigns or community mobilizations **COVID-19**:Coronavirus disease 2019 vaccine

| **Table S3 Details of risk of bias assessment for 5 RCT studies** | | | | | | | |
| --- | --- | --- | --- | --- | --- | --- | --- |
| **Study** | **Risk of bias domains** | | | | | | **Overall bias** |
|  | Randomization process | Selection of participants | Deviation of intended intervention | Missing data | Outcome measurement | Selection of reported results |  |
| Ma et al. (2018) [45] | Some concerns | High | High | High | Low | Low | High |
| Paskett et al. (2016) [52] | High | Low | Low | Low | Low | High | High |
| More et al. (2017) [60] | Low | Low | Low | Low | Low | Low | Low |
| Habib et al. (2017) [59] | Low | Low | Low | Low | Low | Low | Low |
| Oyo-Ita et al. (2021) [55] | Low | Low | Some concerns | Low | Low | Low | Moderate |

| **Table S4 Details of risk of bias assessment for12 pre-post studies and 3 on-RCT studies** | | | | | | | | |
| --- | --- | --- | --- | --- | --- | --- | --- | --- |
| **Study** | **Risk of bias domains** | | | | | | | **Overall bias** |
|  | Randomization process | Selection of participants | Classification  of intervention | Deviation from  intended interventions | Missing data | Outcome measurement | Selection of reported results |  |
| Bailey et al. (2011) [53] | Low | Low | Low | Low | Low | Low | Low | Low |
| Levinson et al. (2013) [57] | Low | Low | Low | Low | Low | Low | Low | Low |
| Weir et al. (2018) [42] | Moderate | Moderate | Low | Low | Low | Low | Low | Moderate |
| Abuelo et al. (2014) [58] | Low | Low | Low | Low | Serious | Low | Low | High |
| Ma et al. (2021) [49] | Low | Low | Low | Low | Low | Low | Low | Low |
| Lennon et al. (2019) [41] | Moderate | Low | Low | Low | Serious | Low | Serious | High |
| Findley et al. (2006) [43] | Low | Low | Low | Low | Low | Low | Serious | High |
| Suryadevara et al. (2013) [44] | Low | Low | Low | Low | Low | Low | Low | Low |
| Marquez et al. (2021) [48] | Low | Low | Low | Low | Low | Low | Low | Low |
| Willis et al. (2016) [46] | Moderate | Low | Low | Low | Low | Low | Low | Moderate |
| Lee et al. (2016) [47] | Moderate | Low | Low | Low | Low | Low | Low | Moderate |
| Sanderson et al. (2017) [50] | Moderate | Low | Low | Low | Serious | Low | Low | High |
| Parra-Medina et al. (2015) [51] | Moderate | Low | Low | Low | Serious | Low | Low | High |
| Akwataghibe et al. (2021) [56] | Moderate | Low | Low | Low | Low | Low | Low | Moderate |
| Bawa et al.(2018) [54] | Low | Low | Low | Low | Low | Low | Low | Low |

**Table S5. The levels of evidence strength**

| **Level** | **Strength assessment** |
| --- | --- |
| 1 Strong evidence | Pooled results derived from three or more studies, including a minimum of two high-quality studies that were statistically homogenous (I^2^ > .05); may be associated with a statistically significant or non-significant pooled result. |
| 2 Moderate evidence | Statistically significant pooled results derived from multiple studies that were statistically heterogeneous (*P* <.05), including at least one high-quality study, or from multiple moderate- or low-quality studies that were statistically homogenous (*P*> .05). |
| 3 Limited evidence | Results from one high-quality study or multiple moderate- or low-quality studies that are statistically heterogeneous (*P*< .05). |
| 4 Very limited evidence | Results from one moderate- or low-quality study. |
| 5 Conflicting evidence | Pooled results that are not significant and derived from multiple studies, regardless of quality, that are statistically heterogeneous (*P*<.05, i.e., inconsistent). |
| 6 No evidence | No studies were identified. |

##


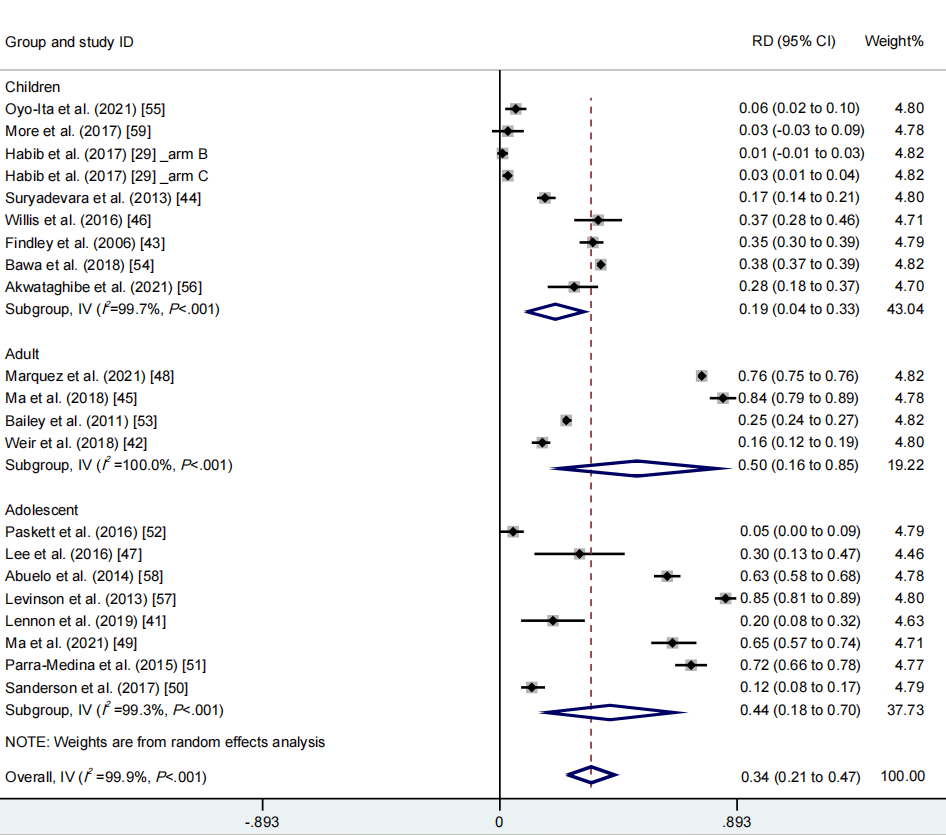


### Figure S1 Subgroup analysis of the effects on vaccination rates by age groups

### (N= 20 studies)


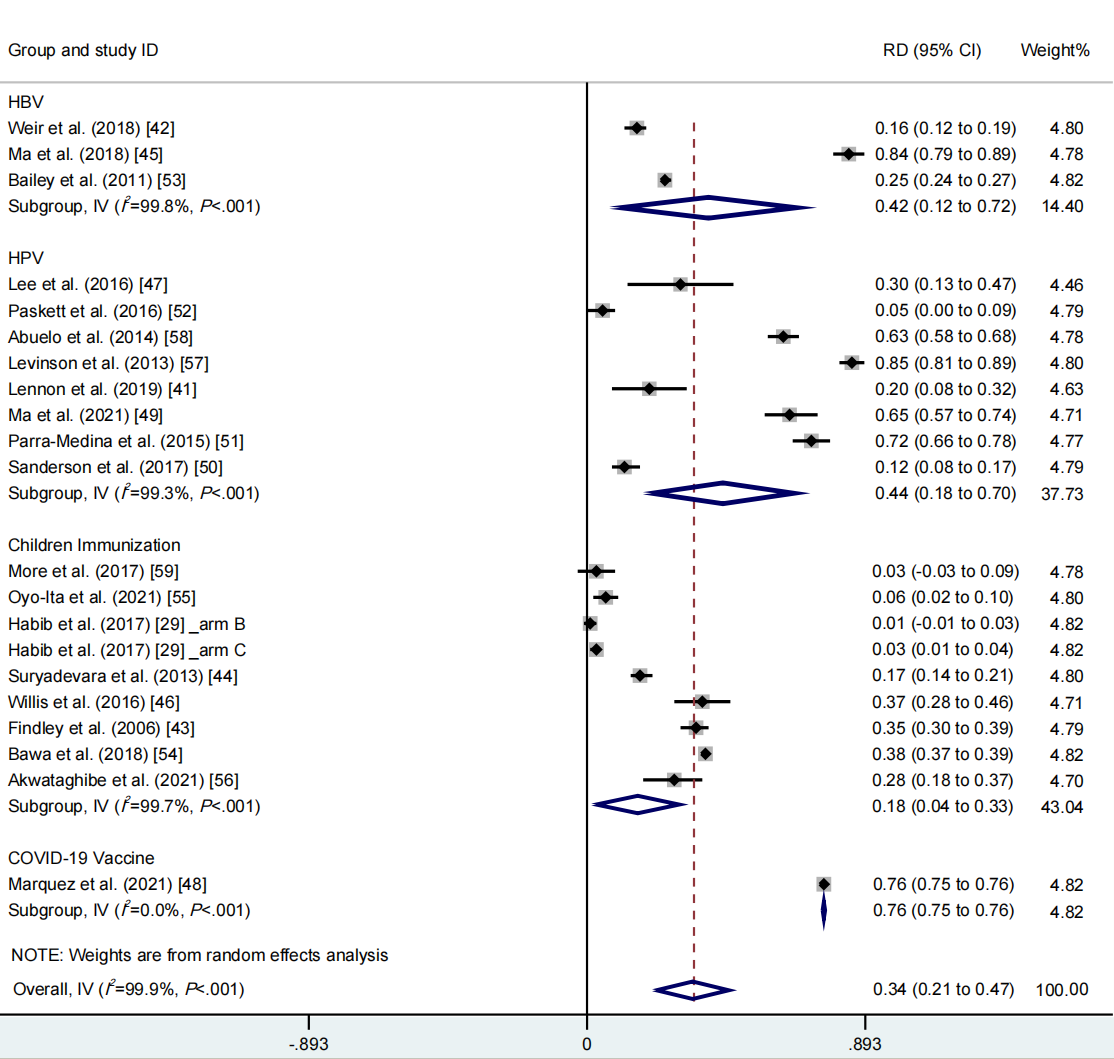


## Figure S2 Subgroup analysis of the effects on vaccination rates by vaccine types

## (N= 20 studies)

Abbreviation: **HPV**: human papillomavirus vaccine **HBV**: hepatitis B virus vaccine **COVID-19**: Coronavirus disease 2019 vaccine


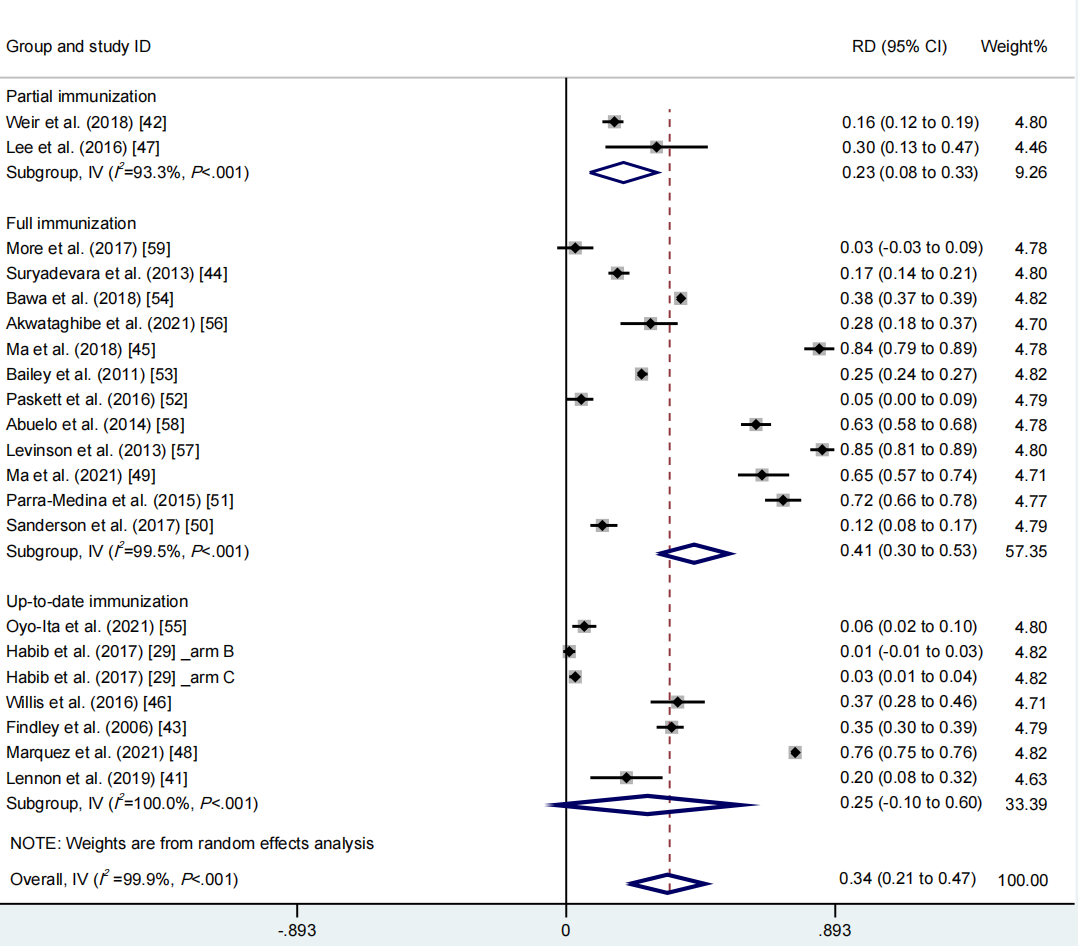


**Figure S3 Subgroup analysis of the effects on vaccination rates by** **immunization definitions**

### (N= 20 studies)


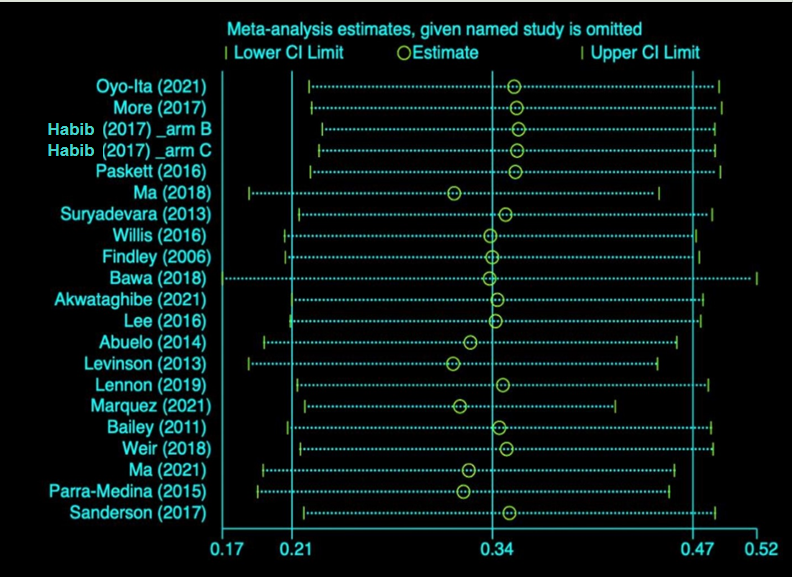
**Figure S4 Sensitivity analysis for the effects of community engagement on vaccination rates in the**

### pre-post intervention effect analysis (N= 20 studies)

Abbreviation: **CI**: confidence interval


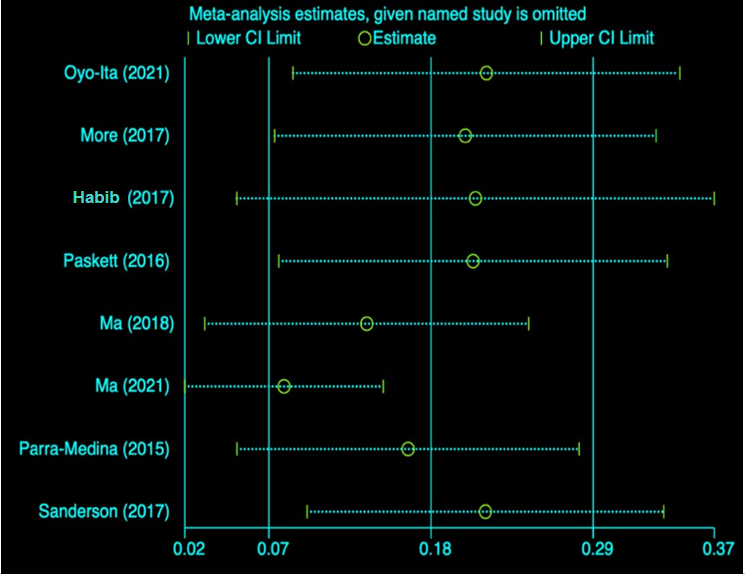


### Figure S5 Sensitivity analysis for the effects of community engagement on vaccination rates in the intervention-control comparison effect analysis (N=8 studies)

Abbreviation: **CI**: confidence interval


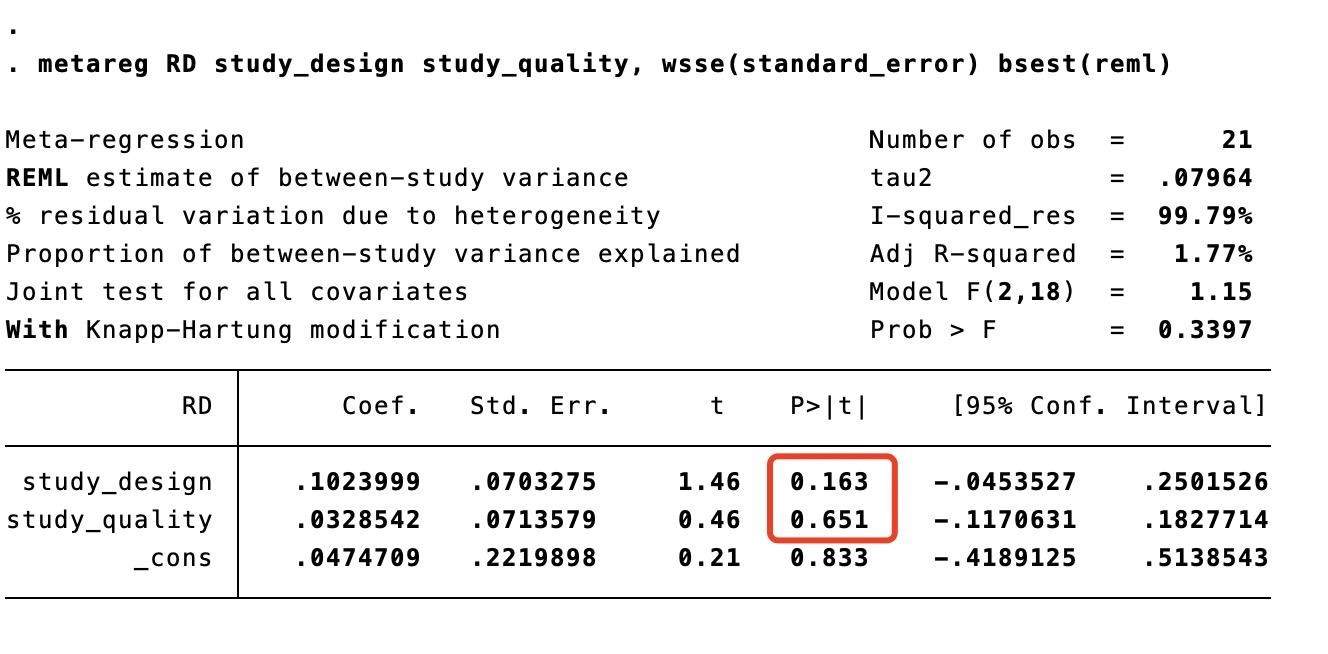


### Figure S6 Meta-regression analysis for the effects of community engagement on vaccination rates in the pre-post intervention effect analysis (N= 20 studies)


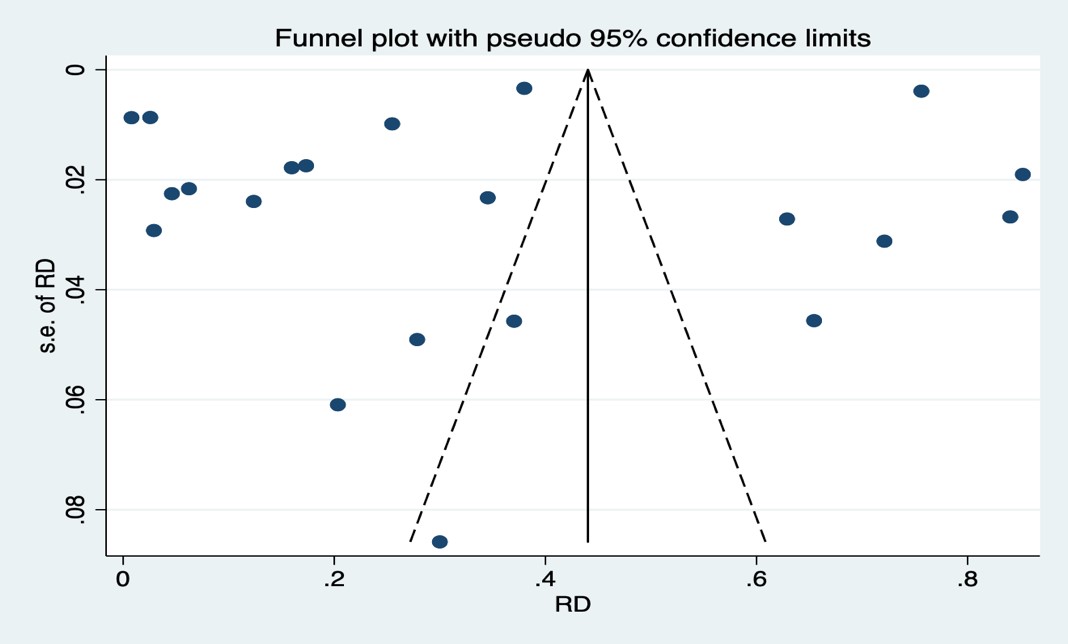


### Figure S7 Funnel plot of publication bias in the effects of community engagement on vaccination rates in the

### pre-post intervention effect analysis (N= 20 studies)


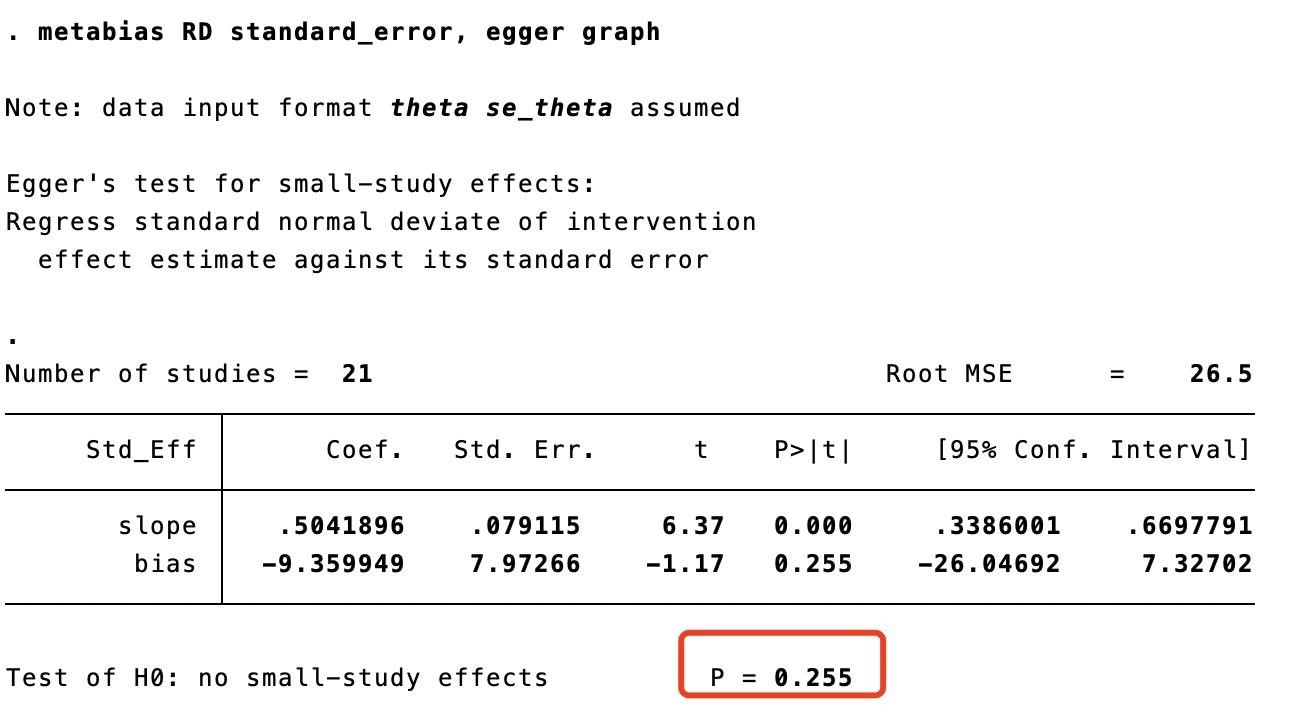


**Figure S8 Egger test of publication bias in the effects of community engagement on vaccination rates in the**

### pre-post intervention effect analysis (N= 20 studies)
